# β cell ER stress and the implications for immunogenicity in type 1 diabetes

**DOI:** 10.3389/fcell.2015.00067

**Published:** 2015-10-27

**Authors:** Meghan L. Marré, Eddie A. James, Jon D. Piganelli

**Affiliations:** ^1^Division of Pediatric Surgery, Department of Surgery, Children's Hospital of Pittsburgh, University of PittsburghPittsburgh, PA, USA; ^2^Benaroya Research Institute at Virginia MasonSeattle, WA, USA

**Keywords:** type 1 diabetes, β cell, ER stress, post-translational modification, neo-antigen, autoimmunity

## Abstract

Type 1 diabetes (T1D) is a chronic autoimmune disease characterized by hyperglycemia due to progressive immune-mediated destruction of insulin-producing pancreatic islet β cells. Although many elegant studies have identified β cell autoantigens that are targeted by the autoimmune response, the mechanisms by which these autoantigens are generated remain poorly understood. Normal β cell physiology includes a high demand for insulin production and secretion in response to dynamic glucose sensing. This secretory function predisposes β cells to significantly higher levels of endoplasmic reticulum (ER) stress compared to nonsecretory cells. In addition, many environmental triggers associated with T1D onset further augment this inherent ER stress in β cells. ER stress may increase abnormal post-translational modification (PTM) of endogenous β cell proteins. Indeed, in other autoimmune disorders such as celiac disease, systemic lupus erythematosus, multiple sclerosis, and rheumatoid arthritis, abnormally modified neo-antigens are presented by antigen presenting cells (APCs) in draining lymph nodes. In the context of genetic susceptibility to autoimmunity, presentation of neo-antigens activates auto-reactive T cells and pathology ensues. Therefore, the ER stress induced by normal β cell secretory physiology and environmental triggers may be sufficient to generate neo-antigens for the autoimmune response in T1D. This review summarizes what is currently known about ER stress and protein PTM in target organs of other autoimmune disease models, as well as the data supporting a role for ER stress-induced neo-antigen formation in β cells in T1D.

## Introduction

Type 1 diabetes (T1D) is a chronic autoimmune disease in which insulin-producing pancreatic islet β cells are targeted and destroyed by autoreactive immune cells. Autoimmune recognition of β cells initiates processes that result in loss of β cell mass and the decline of insulin-mediated control of blood glucose levels. Eventually, the remaining β cells become insufficient to maintain normal blood glucose levels, due to reduced β cell numbers and/or to reduced insulin secretion, and chronic hyperglycemia and T1D ensue.

Given the autoimmune mechanisms of β cell destruction, a major underlying risk factor for T1D is a genetic predisposition to autoimmunity. T1D is a polygenic disease, with many genetic loci associated with disease onset. For example, polymorphisms and variants in many genes related to innate and adaptive immune cell function increase susceptibility to autoimmunity, likely by causing failure of central and peripheral immune tolerance mechanisms. With respect to central tolerance, human leukocyte antigen (HLA), which is the genetic variable with the greatest association to T1D onset (Todd et al., 1987; Dorman et al., 1990; Luca et al., 2008), shapes the adaptive immune repertoire by determining which T cells survive thymic maturation and selection. Under normal circumstances, T cells that respond too strongly to self-peptides presented by HLA are deleted or inactivated (Hogquist and Jameson, 2014). In individuals expressing autoimmune-prone polymorphisms within the HLA gene locus, these central tolerance mechanisms fail, permitting autoreactive T cells to mature, and exit the thymus (Fan et al., 2009; Geenen, 2012). With respect to peripheral tolerance, gene variants at other loci such as protein tyrosine phosphatase, non-receptor type 22 (PTPN22) may accelerate T1D onset through mechanisms that have not been fully elucidated (Pociot and McDermott, 2002; Bottini et al., 2004; Luca et al., 2008; Wallis et al., 2009). For example, some studies suggest that, in the context of genetic predisposition to autoimmunity, incomplete antigen presenting cell (APC) maturation may contribute to T1D progression. These immature APCs do not respond normally to growth factors (Serreze et al., 1993) or to inflammatory stimuli (Serreze et al., 1993; Piganelli et al., 1998). As a result, these APCs exhibit defective antigen processing and presentation that activate autoreactive T cells, but do not trigger tolerogenic mechanisms. Such failure in peripheral immune tolerance may exacerbate T1D pathology. However, as stated above, the precise mechanisms by which many of these genetic variants contribute to T1D remain unknown.

Although genetic predisposition is strongly associated with T1D progression, many epidemiological factors suggest that genetic predisposition is not sufficient to drive pathology. First, only a small portion of individuals with HLA predisposition actually progress to T1D (Knip et al., 2005). Second, monozygotic twins demonstrate relatively low concordance for T1D onset (Barnett et al., 1981; Verge et al., 1995). Third, the incidence of T1D is increasing at a rate that cannot be supported by genetic predisposition alone (Onkamo et al., 1999; Gale, 2002; DIAMOND Project Group, 2006). Finally, the age of onset and rate of progression of T1D vary greatly among patients. Together, these data support a role for environmental factors in triggering T1D onset and affecting progression. Among the many environmental triggers associated with T1D onset are viral infection (Atkinson et al., 1994; Horwitz et al., 1998, 2004; Hiemstra et al., 2001; Härkönen et al., 2002; Schulte et al., 2010), β cell exposure to chemicals (Like and Rossini, 1976; Rossini et al., 1977; Takasu et al., 1991a) or reactive oxygen species (ROS) (Piganelli et al., 2002; Tse et al., 2010; Delmastro and Piganelli, 2011; Delmastro-Greenwood et al., 2014), dysglycemia (Sosenko et al., 2009), and inflammation (Mandrup-Poulsen et al., 1987; Held et al., 1990; Jiang and Woda, 1991). Each of the environmental triggers listed here can cause β cell endoplasmic reticulum (ER) stress, suggesting that ER stress may be a common factor in disease onset. However, whether these environmental factors share common pathways to T1D remains unknown.

To understand how these factors lead to the progression of T1D, scientists have studied the non-obese diabetic (NOD) mouse. Mice of this strain develop spontaneous autoimmune diabetes with many similarities to human T1D. These similarities include genetic susceptibility at the HLA locus and other loci, and intra-islet infiltration of immune cells resulting in β cell destruction (Tochino, 1987; Leiter, 1989; Driver et al., 2012). Seminal studies with this mouse model have identified many β cell antigens targeted by the autoimmune response. These murine autoantigens include preproinsulin (Wegmann et al., 1994), glutamic acid decarboxylase (GAD65) (Tisch et al., 1993), islet-specific glucose-6-phosphatase catalytic subunit-related protein (IGRP) (Lieberman et al., 2003), chromogranin A (CHgA) (Stadinski et al., 2010), islet amyloid polypeptide (IAPP) (Delong et al., 2011), zinc transporter 8 (ZnT8) (Nayak et al., 2014), and 78 kDa glucose-regulated protein (GRP78) (Rondas et al., 2015). With the exception of GRP78, these proteins are also confirmed autoantigens in human T1D (Baekkeskov et al., 1990; Keller, 1990; Gorus et al., 1992; Yang et al., 2006; Wenzlau et al., 2007; Gottlieb et al., 2014) along with additional autoantigens found in humans but not yet identified in NOD mice such as tyrosine phosphatase-like insulinoma antigen 2 (IA-2) and IA-2β [also known as phosphatase homolog of granules from rat insulinomas (phogrin)] (Bonifacio et al., 1995; Lan et al., 1996), and islet cell autoantigen 69 (ICA69) (Pietropaolo et al., 1993). However, the precise mechanisms by which these β cell proteins come to be recognized and targeted by the autoimmune response in T1D remain unknown. Recent evidence suggests that some of these proteins undergo post-translational modification (PTM), generating “neo-antigens” with increased immunogenicity (Dunne et al., 2012). But whether such PTMs occur in the β cell, and what cellular processes might give rise to these PTMs in the β cell, remain unknown.

Here, we discuss cellular conditions (both physiological and pathological) that lead to protein PTM. We also review what is currently known about PTM and neo-antigen generation in target organs of other autoimmune disease models. Finally, we review the evidence supporting a role for ER stress-induced PTM in neo-antigen formation in β cells in T1D.

## ER stress activates the unfolded protein response

The ER is the organelle primarily responsible for folding and PTM of membrane-bound and secreted proteins. To accomplish these tasks, the ER lumen contains the necessary factors to support proper protein folding including molecular chaperones, ATP, an oxidizing environment to support disulfide bond formation, and millimolar concentrations of calcium (Ca^2+^) (Gething and Sambrook, 1992). Proteins that are properly folded exit the ER and continue toward their intended intra- or extra-cellular locations. However, improperly folded proteins remain in the ER and, when too many misfolded proteins accumulate, ER homeostasis is disrupted and ER stress ensues. ER stress activates the cytoprotective unfolded protein response (UPR), which acts to relieve ER stress and restore homeostasis by two mechanisms (Hetz, 2012). First, UPR signaling temporarily inhibits the synthesis of new non-chaperone proteins to prevent further burdening the ER machinery. Second, UPR signaling increases the expression of protein chaperones to aid in the folding of the accumulated misfolded proteins in the ER lumen.

**Figure 1:**
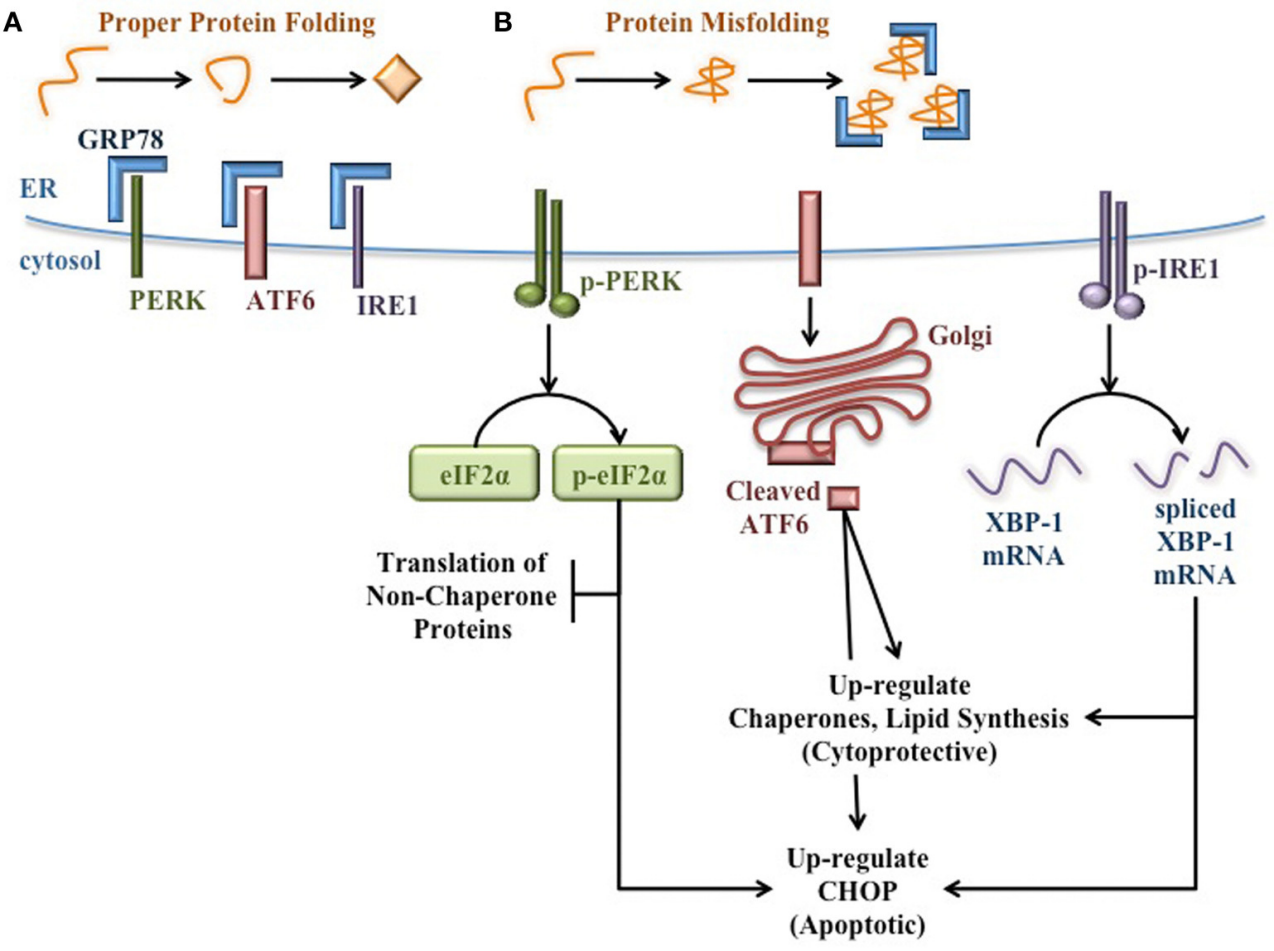
**Signaling pathways of the unfolded protein response**. **(A)** When protein folding proceeds normally, the protein sensors of ER stress (PERK, ATF6, and IRE1) are bound and held in their inactive state by GRP78. **(B)** When misfolded proteins accumulate in the ER lumen, GRP78 binds misfolded proteins, thereby releasing the protein sensors of ER stress and allowing for the activation of the cytoprotective UPR. PERK autophosphorylates in *trans*, then activates eIF2α by phosphorylation to attenuate translation of additional non-chaperone proteins. ATF6 translocates to the Golgi apparatus and is cleaved to yield a transcription factor that up-regulates the expression of molecular chaperones to aid in the folding of accumulated proteins in the ER. IRE1 autophosphorylates in *trans* and splices XBP-1 mRNA. The spliced mRNA encodes a transcription factor that up-regulates the expression of additional molecular chaperones and UPR proteins to relieve ER stress. If ER stress is too great or prolonged, the UPR induces expression of pro-apoptotic proteins such as CHOP.

During normal ER homeostasis, the chaperone GRP78 [also known as binding immunoglobulin protein (BiP)] binds three protein sensors of ER stress that reside in the ER membrane: protein kinase RNA (PKR)-like ER kinase (PERK), activating transcription factor 6 (ATF6), and inositol-requiring protein 1 (IRE1) (Bertolotti et al., 2000; Shen et al., 2002). Interaction with GRP78 keeps these proteins inactive and thereby inhibits the UPR (Figure 1A). However, when misfolded proteins accumulate in the ER, GRP78 releases these protein sensors to bind exposed hydrophobic residues in unfolded proteins. Once free from GRP78, each protein sensor initiates a signaling cascade of the UPR. PERK oligomerizes and becomes activated through autophosphorylation in *trans*. Activated PERK then phosphorylates the α subunit of translation initiation factor 2 (eIF2α) to attenuate mRNA translation and reduce the protein burden in the ER (Harding et al., 2000a,b). ATF6 translocates to the Golgi apparatus where it is cleaved to yield a transcription factor that initiates new chaperone synthesis to aid with folding of accumulated misfolded proteins (Haze et al., 1999). IRE1 oligomerizes and autophosphorylates in *trans*, enabling its endonuclease capability. IRE1 then splices X-box binding protein 1 (XBP-1) mRNA (Yoshida et al., 2001), which encodes a transcription factor that regulates proteins involved in relieving ER stress such as chaperones (Lee et al., 2003) and proteins involved in lipid synthesis to increase ER volume (Sriburi et al., 2004). Through these three signaling cascades, the UPR attempts to reduce ER stress and prevent stress-induced apoptosis (Figure 1B).

However, if the burden of unfolded proteins and the subsequent ER dysfunction are too great or too prolonged, these cytoprotective functions of the UPR fail. Under these conditions, pro-apoptotic signaling pathways become activated and ultimately lead to death of the affected cell (Figure 1B). For example, the UPR induces C/EBP Homologous Protein (CHOP) expression (Wang et al., 1996), which increases ROS-mediated mitochondrial apoptosis signaling pathways (Zinszner et al., 1998; McCullough et al., 2001).

Thus, ER stress and the UPR have significant effects on cellular function and viability. Even in cells that return to homeostasis following UPR activation, ER stress and dysfunction still have consequences. For example, ER stress often results in the release of Ca^2+^ from the ER lumen to the cytosol. Since high Ca^2+^ concentrations are necessary for protein folding, this efflux of Ca^2+^ negatively affects ER function. Second, ER stress and dysfunction lead to abnormal protein folding and PTM, affecting protein function. Therefore, ER stress, even when temporary, may have important effects on cellular function and physiology.

## ER stress is a consequence of normal β cell physiology

All cells undergo some degree of ER stress and activate the UPR in response to improper protein folding or during times of increased protein translation. However, professional secretory cells are uniquely susceptible to ER stress as a result of their normal physiology. In addition to proteins for cellular maintenance, secretory cells are burdened with synthesizing the proteins to be secreted and the proteins required for functional secretory pathways. Thus, the demands of protein translation and folding in the ER of secretory cells are significantly greater than in nonsecretory cells. Although secretory cells contain a more fully developed ER with additional chaperones to compensate for this demand (Shimizu and Hendershot, 2009), the increased ER burden leads to increased occurrence of ER stress.

β cells, like all professional secretory cells, naturally undergo high levels of ER stress as a result of their normal secretory physiology (Araki et al., 2003a; Lipson et al., 2006a,b; Wu and Kaufman, 2006; Fonseca et al., 2007; Ortsäter and Sjöholm, 2007; Eizirik et al., 2008; Volchuk and Ron, 2010; Kim et al., 2012; Teodoro et al., 2012). Indeed, β cells undergo significant ER stress during postprandial glucose-stimulated insulin synthesis (Lipson et al., 2006a,b). β cells increase translation of preproinsulin by 50-fold in response to heightened blood glucose concentrations, reaching a production rate of 1 million molecules of preproinsulin per minute (Scheuner and Kaufman, 2008). These 1 million molecules flood the ER lumen for folding and disulfide bond formation, causing tremendous ER stress. Such cellular processes of dynamic insulin production and heightened ER stress occur from an early age. In XBP-1 splicing reporter mice, the pancreas was the first tissue to exhibit high levels of ER stress and did so as early as 16 days old post birth (Iwawaki et al., 2004). Therefore, normal insulin-secreting physiology alone significantly increases ER stress in β cells.

**Figure 2:**
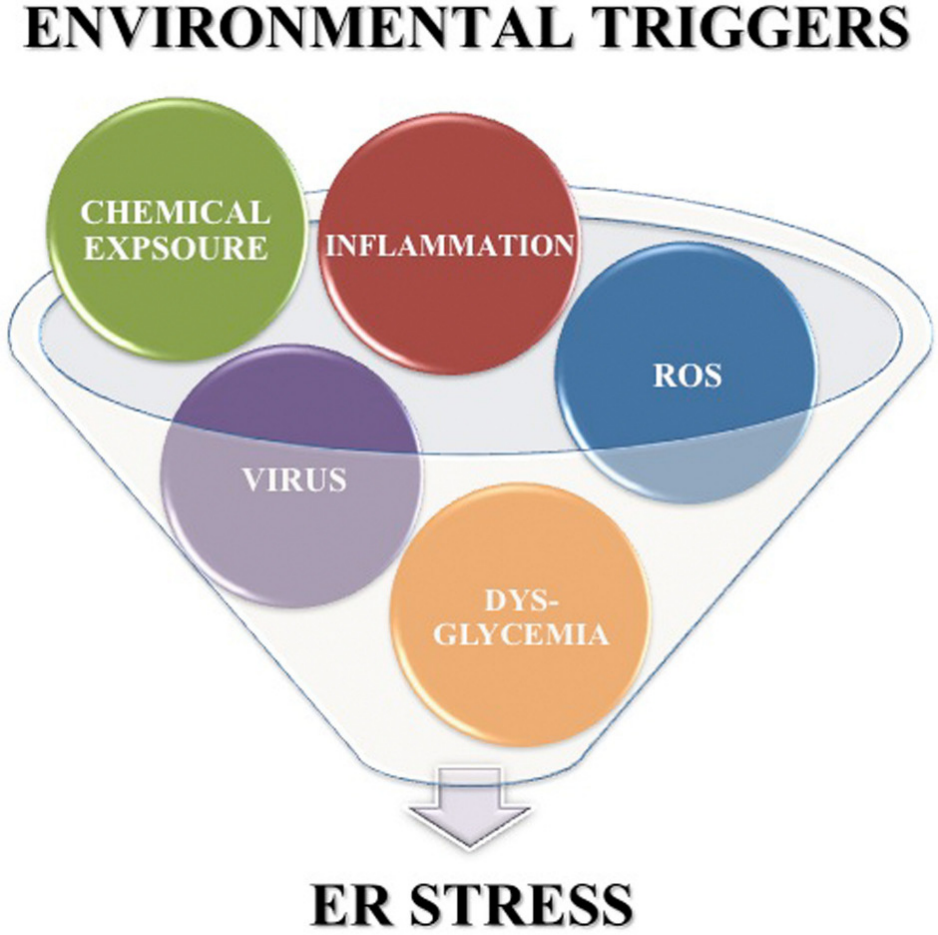
**Environmental triggers associated with T1D exacerbate β cell ER stress**. Environmental factors such as viral infection, chemicals, ROS, dysglycemia, and pancreatic inflammation are associated with onset of T1D. Each of these environmental triggers of T1D also increases β cell ER stress above the inherently high levels induced by normal β cell physiology.

In addition to the high levels of inherent ER stress, many of the putative environmental triggers associated with T1D may further enhance β cell ER stress. First, Coxsackie viral infection disrupts the ER membrane (van Kuppeveld et al., 1997, 2002, 2005) releasing Ca^2+^ from the ER into the cytosol. Second, β cell exposure to chemicals such as streptozotocin and alloxan cause protein ADP-ribosylation (Sandler and Swenne, 1983) and ROS generation (Heikkila et al., 1976; Takasu et al., 1991b; Bedoya et al., 1996), both of which lead to protein misfolding, and also decrease ER lumen Ca^2+^ concentrations (Kim et al., 1994; Park et al., 1995). Third, β cell exposure to ROS from either extracellular or intracellular sources releases Ca^2+^ from the ER lumen into the cytosol (Favero et al., 1995; Xu et al., 1998; Görlach et al., 2006). Also, dysglycemia leads to increased glucose sensing that, as discussed above, significantly increases insulin production and secretion (Scheuner and Kaufman, 2008). Finally, pancreatic inflammation and cytokine exposure activates c-jun N-terminal (JNK) mitogen-activated protein (MAP) kinase signaling pathways (Wang et al., 2009; Lee et al., 2011). The cellular effects of each environmental trigger exacerbate β cell ER stress. Therefore, although the precise mechanisms by which these environmental triggers accelerate T1D may vary, all the factors listed here can increase β cell ER stress above the normal physiological levels. Therefore, heightened ER stress may be a common factor in early T1D pathogenesis (Figure 2).

ER stress and diabetes have been linked in both human and mouse studies. In studies of human islets, ER stress markers were increased in islets of T1D patients compared to islets of nondiabetic controls (Marhfour et al., 2012). In the Akita mouse model, the *Ins*2^*C*96*Y*^ mutation prevents the formation of a crucial disulfide bond leading to misfolded insulin (Ron, 2002) and high ER stress in these β cells (Ron, 2002; Araki et al., 2003b; Nozaki et al., 2004). This ER stress leads to β cell apoptosis through the activation of CHOP signaling pathways (Oyadomari et al., 2002; Ron, 2002). However, inhibition of CHOP-mediated apoptosis merely delays, but does not halt, β cell loss and disease onset (Oyadomari et al., 2002). These data suggest that apoptosis may not be the only mechanism by which ER stress causes β cell death and diabetes.

## ER stress alters Ca^2+^ concentrations in the ER lumen and cytosol

In addition to folding and PTM of proteins, the ER is an important organelle for the maintenance of intracellular Ca^2+^ homeostasis. The ER contains the largest intracellular store of Ca^2+^ and is an important source of Ca^2+^ necessary for regulating a variety of cellular functions both in the ER lumen and in the cytosol (Meldolesi and Pozzan, 1998).

**Figure 3:**
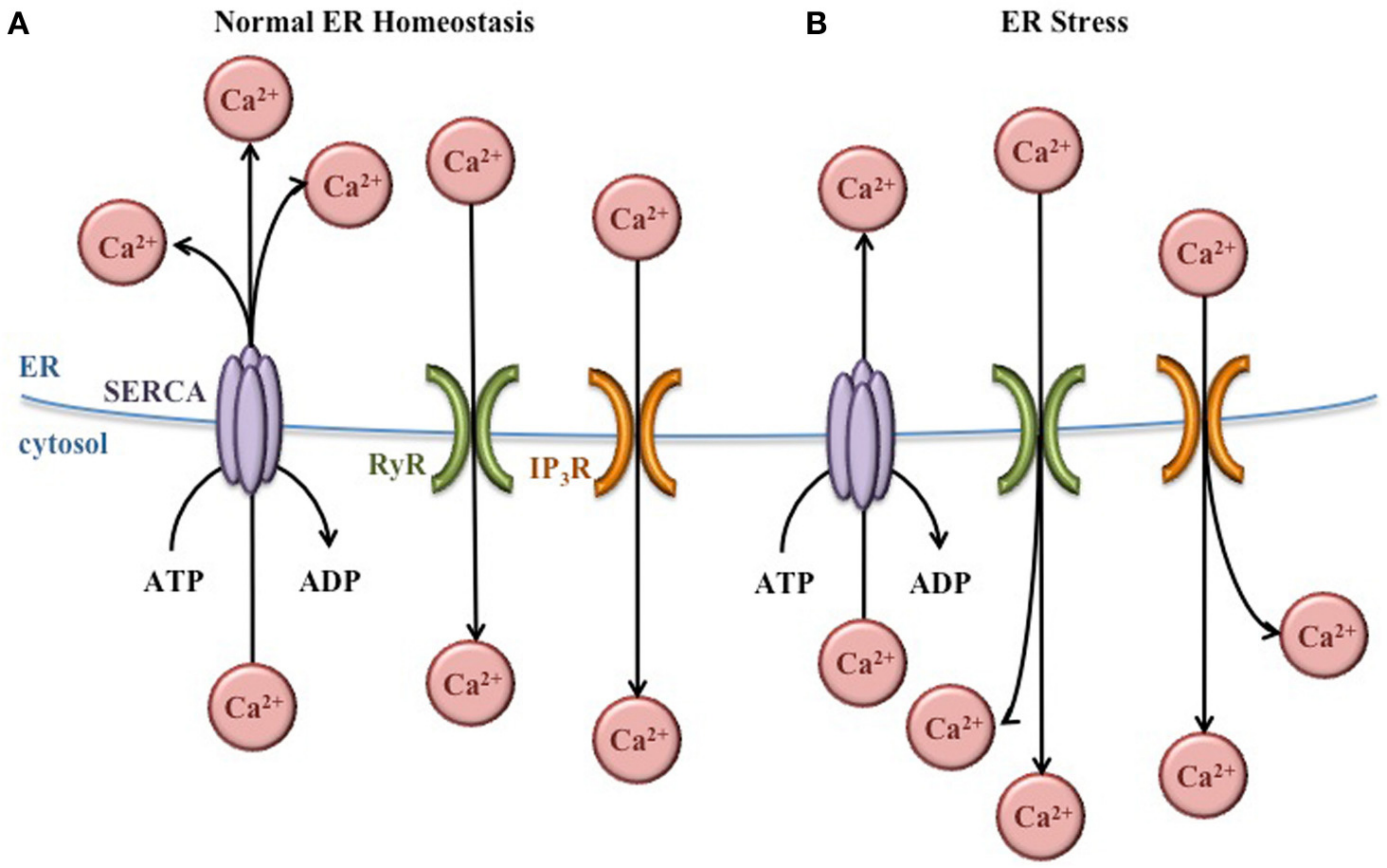
**Regulation of ER Ca^2+^ concentrations**. **(A)** Under normal conditions, Ca^2+^ concentrations are higher in the ER lumen than in the cytosol. This balance is maintained by SERCA pumps that bring Ca^2+^ into the ER lumen, and Ca^2+^ channels (RyR and IP_3_R) that release Ca^2+^ into the cytoplasm as needed for normal cellular signaling. **(B)** During ER stress, the Ca^2+^ gradient across the ER membrane is disturbed, leading to Ca^2+^ release from the ER and increased Ca^2+^ concentrations in the cytoplasm.

Within the ER lumen, high concentrations of Ca^2+^ are important for proper protein folding. Many molecular chaperones, including GRP78, are Ca^2+^-dependent (Ma and Hendershot, 2004). In addition, the proteins that facilitate the formation of disulfide bonds [protein disulfide isomerases (PDI)] also require Ca^2+^ (Nigam et al., 1994). To maintain the high concentration Ca^2+^ necessary for ER function, sarco/endoplasmic reticulum Ca^2+^ ATPases (SERCA) pumps in the ER membrane actively transport Ca^2+^ from the cytosol into the ER lumen (Figure 3). These pumps are regulated by existing concentrations of Ca^2+^ in the lumen to prevent ER Ca^2+^ stores from rising too high. Inhibition of these SERCA pumps prevents the movement of Ca^2+^ into the ER, decreasing the function of molecular chaperones and PDI, and increasing the burden of misfolded protein in the ER (Mekahli et al., 2011).

In the cytosol, Ca^2+^ plays important roles in a variety of cellular functions including metabolism, vesicular trafficking, secretion, transcription, and apoptosis (Berridge et al., 2000). Ca^2+^ channels in the ER membrane such as ryanodine-receptor (RyR) and inositol 1,4,5-trisphosphate receptor (IP_3_R) release Ca^2+^ from the ER lumen into the cytosol according to its chemical gradient (Figure 3). Like the SERCA pumps, the function of these channels is regulated to prevent depletion of the ER Ca^2+^ concentrations (Mekahli et al., 2011).

In spite of the regulation of SERCA pumps and Ca^2+^ channels, the normal Ca^2+^ gradient across the ER membrane is altered during ER stress, leading to decreased Ca^2+^ in the ER and increased Ca^2+^ in the cytosol. These changes in Ca^2+^ concentrations have important effects for the cell. The ER chaperones and PDI necessary for proper protein folding depend on Ca^2+^, so this imbalance exacerbates ER stress and further activates the UPR. In addition, increased cytosolic Ca^2+^ can cause apoptosis. For instance, Ca^2+^ release from the ER activates the ER-associated procaspase 12 (murine) or procaspase 4 (human), which initiate the caspase cell death pathway (Nakagawa et al., 2000; Hitomi et al., 2004). Also, the Ca^2+^-dependent ER chaperone calreticulin (Michalak et al., 2009) activates caspase 3- and cytochrome c-dependent apoptosis pathways when ER Ca^2+^ concentrations decrease (Nakamura et al., 2000). Furthermore, increased cytosolic Ca^2+^ activates enzymes such as calpain and calciuneurin which activate mitochondria-dependent signaling cascades that ultimately lead to cellular apoptosis (Nakagawa and Yuan, 2000; Gil-Parrado et al., 2002; Kim et al., 2002; Hajnóczky et al., 2003).

Therefore, the maintenance of Ca^2+^ homeostasis is crucial for cellular health and function. Disruption of this Ca^2+^ gradient across the ER membrane has major consequences for ER function and cellular viability.

## Increased cytosolic Ca^2+^ activates post-translational modification enzymes

While the of activation apoptotic signaling pathways usually requires prolonged ER stress and disrupted Ca^2+^ gradients, other cytosolic Ca^2+^-depenent enzymes are activated in response to more transient ER stress and heightened cytosolic Ca^2+^ concentrations. In particular, two families of Ca^2+^-dependent PTM enzymes are activated during ER stress. The activation of these enzymes has important implications for the proteins being folded in the ER.

### Tissue transglutaminase 2

Tissue transglutaminase 2 (Tgase2) is a ubiquitously expressed Ca^2+^-dependent PTM enzyme that resides in the cytosol (Lesort et al., 1998). Tgase2 becomes activated when Ca^2+^ concentrations in the cytosol rise above normal physiological levels. Indeed, Tgase2 activity requires Ca^2+^ concentrations above what is necessary for normal cellular signaling. As such, Tgase2 usually becomes activated only when cellular homeostasis is disrupted, such as when Ca^2+^ is released from the ER during ER stress (Ientile et al., 2007; Kojima et al., 2010; Wilhelmus et al., 2011; Kuo et al., 2012; Verhaar et al., 2012). Once active, Tgase2 translocates to several intra- and extra-cellular compartments (Park et al., 2010) including the ER (Orru et al., 2003; Wilhelmus et al., 2011; Verhaar et al., 2012) and secretory granules (Russo et al., 2013), to modify proteins by two mechanisms (Facchiano et al., 2006): first, Tgase2 forms ε (γ-glutamyl) isopeptide bonds between gluatmine and lysine residues that crosslink proteins, and second, Tgase2 facilitates the deamidation of glutamine. PTM of proteins by Tgase2 is important for a variety of normal cellular processes (Fesus and Piacentini, 2002; Gundemir et al., 2012). For example, Tgase2 modifies caspase 3 (Yamaguchi and Wang, 2006) and mitochondrial proteins (Fok and Mehta, 2007) to regulate apoptosis, nuclear proteins to regulate gene expression (Ballestar et al., 1996; Lesort et al., 1998; Han and Park, 2000), and extracellular matrix protein to promote cell adhesion (Gaudry et al., 1999; Akimov et al., 2000) and wound healing (Haroon et al., 1999; Stephens et al., 2004; Verderio et al., 2004).

### Peptidylarginine deiminase

Peptidylarginine deiminases (PAD) are another family of Ca^2+^-dependent PTM enzymes that reside in the cytosol (Vossenaar et al., 2003b). Of the five mammalian isoforms, PAD2 is the most widely expressed, and is the isoform expressed in the pancreas (Takahara et al., 1989). PAD become activated when cytosolic Ca^2+^ concentrations increase to levels 100-fold above normal physiological levels (Takahara et al., 1986; Vossenaar et al., 2003b). When activated, PAD are recruited to various subcellular compartments to modify proteins (Jang et al., 2011). PAD convert arginine to citrulline, which causes a loss of a positive charge in the amino acid sequence (Rogers et al., 1977). This change in charge has significant implications for protein folding, interaction, and function (Tarcsa et al., 1996). PAD play several roles in the context of normal cellular physiology. For example, PAD target IκB kinase gamma (IKKγ) to inhibit nuclear factor kappa-light-chain-enhancer of activated B cells (NF-κB) activation (Lee et al., 2010), target vimentin to regulate cytoskeletal disassembly (Inagaki et al., 1989), and are important in the formation of neutrophil extracellular traps (NET) (Li et al., 2010).

## Ca^2+^-dependent PTM generates neo-antigens

Although PTMs are important in normal cellular signaling and physiology, PTM of proteins may contribute to autoimmune disorders. If proteins are modified differently in peripheral tissues than in the thymus, the modified peripheral proteins may act as neo-antigens for which there is no immune tolerance (Doyle and Mamula, 2012). Indeed, a variety of PTMs are implicated in the pathology of several autoimmune diseases (Table 1). Importantly, many neo-antigens are formed through PTM by the Ca^2+^-dependent enzymes Tgase2 and PAD. For example, Tgase2 activity is significantly elevated in celiac disease patients (Bruce et al., 1985). Tgase2 forms intermolecular ε (γ-glutamyl) isopeptide bonds, generating dimers of itself and gliadin as well as oligomers of gliadin (Molberg et al., 1998; Fleckenstein et al., 2004). These complexes are recognized by the immune system as neo-antigens, giving rise to increased T cell responses (Molberg et al., 1998) and anti-Tgase2 antibody production (Dieterich et al., 1997). These immune responses exacerbate the inflammatory conditions in the gut (Halttunen and Mäki, 1999; Barone et al., 2007). Also, in multiple sclerosis, citrullination of myelin basic protein forms a neo-antigen to which T cells respond (Martin et al., 1994). This neo-antigen causes disease in experimental autoimmune encephalomyelitis (the mouse model of multiple sclerosis) (Zhou et al., 1995). Finally, in rheumatoid arthritis, patients develop autoantibodies to the citrullinated forms of many proteins (Schellekens et al., 1998; Masson-Bessière et al., 2001; Vossenaar et al., 2003a, 2004; Burkhardt et al., 2005; Kinloch et al., 2005). These autoantibodies are detected in the synovial fluid of rheumatoid arthritis patients at early stages of disease (van Boekel et al., 2002; Vasishta, 2002), suggesting the importance of these PAD-generated neo-antigens for disease progression.

**Table 1:** **Neo-antigens formed by PTM in autoimmune diseases**.

**Disease**	**Autoantigen**	**PTM**	**References**
Celiac disease	Gliadin	Deamidation	Molberg et al., 1998
Collagen-induced arthritis	Type II collagen	Glycosylation	Corthay et al., 1998
		Hydroxylation	Corthay et al., 1998
Multiple Sclerosis/EAE	Myelin basic protein	Acetylation	Zamvil et al., 1986
		Citrullination	Martin et al., 1994
	Myelin oligodendrocyte glycoprotein	Malondialdehyde	Wållberg et al., 2007
	αB-crystallin	Phosphorylation	van Stipdonk et al., 1998
Rheumatoid Arthritis	Filaggrin	Citrullination	Schellekens et al., 1998
	Fibrin	Citrullination	Masson-Bessière et al., 2001
	Fibrinogen	Citrullination	Vossenaar et al., 2003a
	Vimentin	Citrullination	Vossenaar et al., 2004
	Collagen	Citrullination	Burkhardt et al., 2005
	α-Enolase	Citrullination	Kinloch et al., 2005
Systemic lupus erythematosus	Small nuclear ribonucleoprotein particle	Isoaspartylation	Mamula et al., 1999
	70 kd subunit of U1 small nuclear ribonucleoprotein particle	Phosphorylation	Monneaux et al., 2003
	Lupus La protein	Phosphorylation	Coudevylle et al., 2006
	SmD1/SmD3	Methylation	Brahms et al., 2000

Although Tgase2- and PAD-mediated PTMs are known to generate neo-antigens, little research has been conducted regarding the precise mechanisms by which these pathological PTMs arise in the particular cells and tissues targeted in these autoimmune disease models. However, Tgase2 and PAD, as described above, become activated under conditions that cause significantly elevated cytosolic Ca^2+^. The main cause of significantly elevated Ca^2+^ is cellular stress, especially ER stress. Therefore, ER stress may give rise to neo-antigen formation through abnormal Ca^2+^-dependent PTM of endogenous proteins.

## T1D autoantigens exhibit increased immunogenicity after PTM

Although it is well established that PTM of endogenous proteins forms neo-antigens that initiate and exacerbate the autoimmune response in many autoimmune diseases (Table 1), the role of PTM in β cell autoantigen formation long remained unexplored. However, in the last 10 years, many seminal studies have demonstrated that known murine and human β cell autoantigens exhibit greater immunogenicity after PTM (Table 2). For example, T cells from a human T1D patient recognized an oxidized epitope of proinsulin (Mannering et al., 2005). These T cell responses depended on the formation of a vicinal disulfide bond, as replacement of either cysteine with a serine residue abolished T cell responses against this peptide. In addition, PTM by the Ca^2+^-dependent enzymes Tgase2 and PAD increases the immunogenicity of several β cell proteins.

**Table 2:** **Neo-antigens formed by PTM in T1D**.

**Autoantigen**	**PTM**	**References**
Proinsulin	Oxidation	Mannering et al., 2005
CHgA (WE14)	Crosslinking/ Isospeptide Bond	Delong et al., 2012; Gottlieb et al., 2014
Preproinsulin	Deamidation	van Lummel et al., 2014
ICA69	Deamidation	van Lummel et al., 2014
ZnT8	Deamidation	van Lummel et al., 2014
Phogrin	Deamidation	van Lummel et al., 2014
IA-2	Deamidation	van Lummel et al., 2014
IGRP	Deamidation	van Lummel et al., 2014
GAD65	Citrullination	McGinty et al., 2014
	Deamidation	McGinty et al., 2014; van Lummel et al., 2014
GRP78	Citrullination	Rondas et al., 2015

### Chromogranin A

The first β cell autoantigen shown to elicit greater immune responses after Ca^2+^-dependent modification was the WE14 peptide of chromogranin A (CHgA) (Delong et al., 2012). The authors had previously demonstrated that the BDC2.5 diabetogenic CD4^+^ T cell clone recognizes WE14 (Stadinski et al., 2010). However, exceptionally high peptide concentrations were required for full T cell activation. In this study, Delong et al. demonstrated that treatment of WE14 with Tgase2 generated a covalently cross-linked peptide that is preferentially presented to BDC2.5 T cells, thereby increasing proliferation and IFNγ production. In addition, splenocytes isolated from pre-diabetic NOD mice responded more strongly to Tgase2-modified WE14 than to the native peptide. A subsequent study showed that WE14 was recognized by T cells from human T1D patients, and that treatment of WE14 with Tgase2 increased the response elicited from these T cells (Gottlieb et al., 2014). This confirmed the relevance of this modified β cell antigen to human T1D. Together, these studies demonstrated that Tgase2-modification of CHgA contributes to the strong activation of autoreactive immune cells in T1D.

### Preproinsulin

The deamidation of glutamine by Tgase2 also modulates the recognition of β cell antigens. In a recent study, deamidated peptides from many islet proteins were eluted from T1D-associated HLA-DQ proteins (van Lummel et al., 2014). These Tgase2-modified peptides bound more strongly than their unmodified counterparts to HLA-DQ molecules. Among these, a Tgase2-modified peptide from preproinsulin elicited responses from CD4^+^ T cells from a new-onset T1D patient. This study therefore identified novel islet peptides that become neo-antigens through PTM. This study also demonstrated stronger binding to disease-associated HLA molecules as one mechanism by which β cell neo-antigens elicit stronger autoimmune responses.

### GAD65

Another β cell protein shown to elicit greater immune responses after PTM is GAD65. Modification of multiple GAD65 peptides by either Tgase2 (deamidation) or PAD (deimidation) increased immunogenicity (McGinty et al., 2014). These peptides bind MHC class II molecules more strongly than the native peptides. Furthermore, T cells that recognize the modified peptides were present at higher frequencies in human T1D patients than in HLA-matched control subjects. These T cells responded to Tgase2-modified peptides of GAD65 more strongly than to the unmodified peptides and displayed a disease-relevant memory phenotype. These data demonstrated a role for Ca^2+^-dependent PTM in increasing immunogenicity of GAD65 peptides, and further supported a role for PTM-mediated neo-antigen generation in human T1D.

### GRP78

Most recently, citrullinated GRP78 was identified as an autoantigen in diabetic NOD mice (Rondas et al., 2015). CD4^+^ T cells from diabetic NOD mice secreted significantly higher IFNγ in response to citrullinated GRP78 compared to T cells from non-diabetic mice. In addition, new-onset diabetic NOD mice exhibited higher titers of autoantibodies that recognize modified GRP78 compared to age-matched non-diabetic mice. Importantly, these T cell responses and α-GRP78 autoantibodies specifically recognized the ctirullinated peptide, not the native peptide, demonstrating the relevance of PTM to the generation of this neo-antigen. This study, therefore, identified modified GRP78 as a novel autoantigen in the NOD mouse model of T1D.

Together, these studies demonstrate that, as in other autoimmune disorders, PTM enhances the immunogenicity of several known autoantigens in T1D. However, these studies were conducted with synthetic peptides that were modified *in vitro* or designed to mimic modified sequences. Whether the β cell proteins from which these peptides are derived undergo PTM within the β cell remains unknown. In addition, the mechanisms by which Tgase2 and PAD might be activated in the β cell remain undefined. However, as we have discussed here, Tgase2 and PAD are both Ca^2+^-dependent and known to be activated during ER stress. β cells inherently undergo particularly high levels of ER stress, which may be further increased upon exposure to environmental triggers of T1D. This high ER stress may activate Tgase2 and PAD to modify endogenous β cell proteins, generating neo-antigens. Therefore, β cell autoantigens may become immunogenic due to ER stress-induced PTM.

## Conclusion

**Figure 4:**
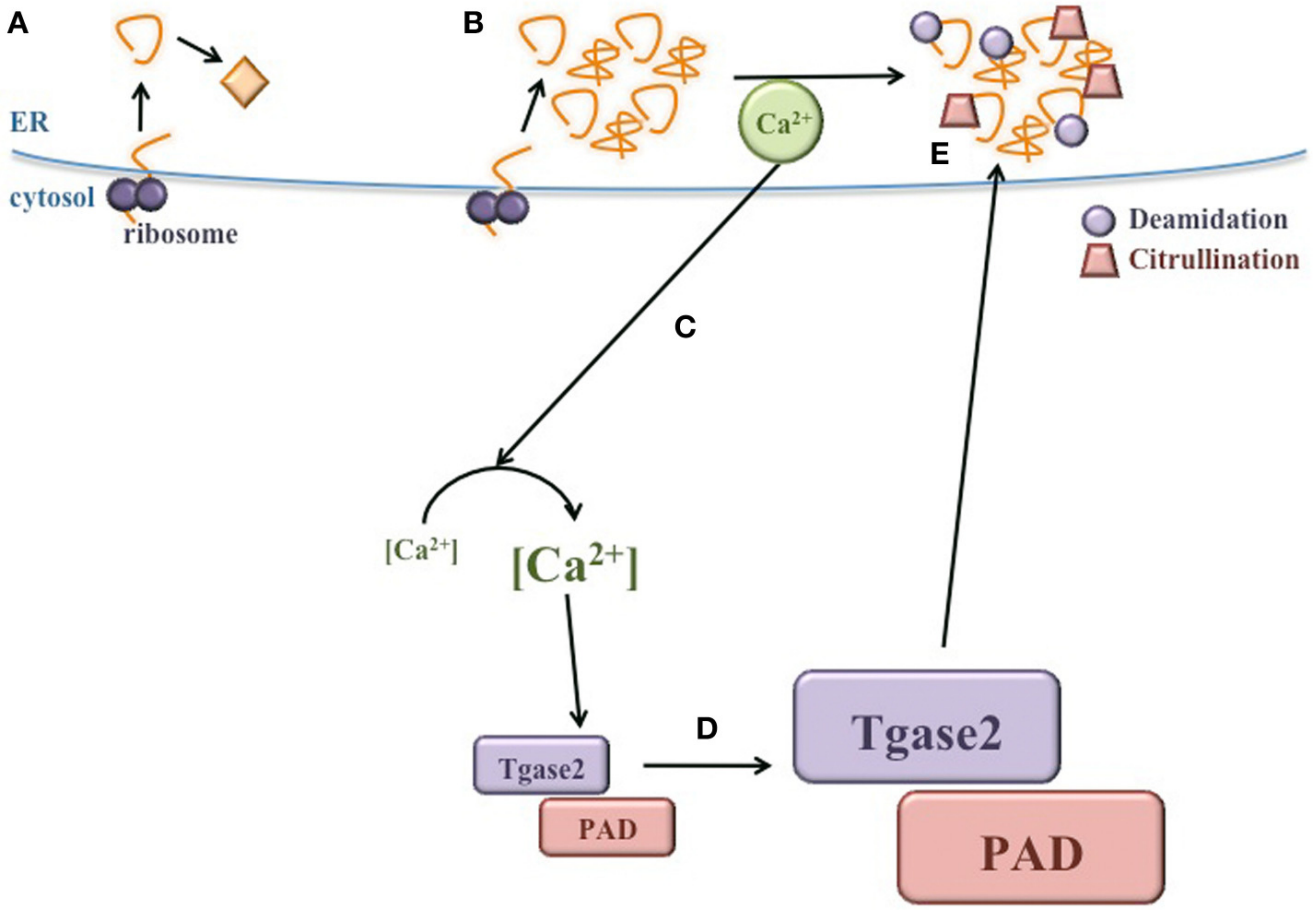
**ER stress induces neo-antigen formation in β cells. (A)** During normal conditions, proteins are translated and properly folded in the ER lumen. **(B)** During ER stress, proper protein folding is inhibited and misfolded proteins accumulate. **(C)** Ca^2+^ is released from the ER, significantly increasing the concentration of cytosolic Ca^2+^. **(D)** Heightened Ca^2+^ in the cytosol increases the activity of PTM enzymes, such as Tgase2 and PAD. **(E)** Activated Tgase2 and PAD modify β cell proteins, generating neo-antigens for the autoimmune response in T1D.

Many elegant and seminal studies have demonstrated that peptides derived from β cell autoantigens become more immunogenic after PTM (Mannering et al., 2005; Delong et al., 2012; Dunne et al., 2012; Gottlieb et al., 2014; McGinty et al., 2014; van Lummel et al., 2014; Rondas et al., 2015). However, the mechanisms by which these neo-antigens are modified in the β cell have not yet been elucidated. Here, we propose that the normal physiology of the β cell, together with the exposure of β cells to a variety of environmental factors, significantly raises ER stress, leading to the release of Ca^2+^ from the ER lumen into the cytosol. This Ca^2+^ flux may activate cytosolic PTM enzymes, which could modify β cell proteins, generating neo-antigens (Figure 4). Because islet β cells are inherently susceptible to high ER stress, these PTMs may occur in all β cells in all individuals. Therefore, T1D onset may not be determined by whether these neo-antigens are generated, but perhaps by genetic predisposition to autoimmunity. Individuals without a genetic predisposition to autoimmunity do not experience a failure of immune tolerance due to central and peripheral mechanisms that maintain immunological tolerance. Thus, the presentation of ER stress-induced modified neo-antigens by APCs may not activate peripheral T cells and T1D may not occur. In contrast, individuals that do harbor genetic predispositions to autoimmunity experience defects in mechanisms of immune tolerance. In these individuals, presentation of modified neo-antigens by APCs could activate autoreactive T cells and cause autoimmune destruction of β cells.

Once the autoimmune response is initiated, the effects of β cell ER stress are magnified. ER stress progressively increases with immune infiltration into the islet (Tersey et al., 2012). Heightened ER stress could lead to increased cytosolic Ca^2+^ and increased activity of Tgase2 and PAD. Recent studies have shown that Tgase2- and PAD-mediated PTMs increase the immunogenicity of peptides derived from known β cell autoantigens (Table 2). Therefore, as β cell ER stress progressively increases, these ever-more active enzymes may modify proteins beyond their physiological targets, including known β cell autoantigens. These neo-antigens could be processed and presented by APCs to T cells in draining lymph nodes. Activated immune cells returning to the islet may further increase β cell ER tress by two mechanisms. First, activated immune cells secrete cytokines that directly increase ER stress. Second, immune-mediated destruction reduces β cell mass, requiring the remaining β cells to produce more insulin per cell and augmenting the ER stress in each β cell. Increased ER stress likely leads to the generation of additional neo-antigens, further fueling the autoimmune response. Therefore, once pathology is initiated in T1D, the cycle of ER stress and neo-antigen generation likely hastens the onset of T1D and continues until the β cell mass is fully destroyed.

The recent studies that have identified modified β cell peptides as neo-antigens have opened important new areas of research in the field of T1D. Additional studies to confirm the cause of increased ER stress in the β cell, and to establish the role of ER stress in the generation of these neo-antigens, will further advance the field. In particular, understanding how these neo-antigens arise in β cells will identify opportunities for therapeutic intervention before the β cell mass is destroyed. For example, therapies that aid in proper protein folding or otherwise reduce ER stress may prevent the formation of neo-antigens. Alternatively, therapeutic agents that promote the degradation of abnormal proteins may remove neo-antigens from β cells. Either therapeutic mechanism may prevent immune-mediated recognition of β cells. Indeed, therapeutic agents that reduce ER stress or degrade misfolded proteins are effective in other disease models (Boyce et al., 2005; Ozcan et al., 2006; Harris and Rubinsztein, 2011; Zode et al., 2011; Bachar-Wikstrom et al., 2012; Hasnain et al., 2012; Engin et al., 2013; Jiang et al., 2015). Similar treatments in T1D models may reduce ER stress-induced neo-antigen formation in β cells, preventing immune destruction of β cells and preventing onset of T1D.

## Author contributions

MM, EJ, and JP contributed to the composition of this manuscript.

## Funding

This work was supported in part by the Juvenile Diabetes Research Foundation (3-PDF-2014-213-A-N to MM, 2-SRA-2014-297-Q-R to EJ, and 2-SRA-2014-296-Q-R to JP), the American Diabetes Association (1-12-BS-161 to JP), and Children's Hospital of Pittsburgh of the UPMC Health System (Cochrane-Weber Endowed Fund to JP).

### Conflict of interest statement

The authors declare that the research was conducted in the absence of any commercial or financial relationships that could be construed as a potential conflict of interest.

## Abbreviations

APC: antigen presenting cell
ATF6: activating transcription factor 6
BiP: binding immunoglobulin protein
Ca^2+^: calcium
CHgA: chromogranin A
CHOP: C/EBP Homologous Protein
EAE: experimental autoimmune encephalomyelitis
eIF2α: α subunit of translation initiation factor 2
ER: endoplasmic reticulum
GAD: glutamic acid decarboxylase
GRP78: 78 kDa glucose-regulated protein
HLA: human leukocyte antigen
IA-2: tyrosine phosphatase-like insulinoma antigen 2
IAPP: islet amyloid polypeptide
ICA69: islet cell autoantigen 69
IGRP: islet-specific glucose-6-phosphatase catalytic subunit-related protein
IKKγ: IκB kinase gamma
IP_3_R, inositol 1, 4: 5-trisphosphate receptor
IRE1: inositol-requiring protein 1
JNK: c-jun N-terminal
MAP kinase: mitogen-activated protein kinase
NET: neutrophil extracellular traps
NF-κB: nuclear factor kappa-light-chain-enhancer of activated B cells
NOD mouse: non-obese diabetic mouse
PAD: peptidylarginine deiminases
PDI: protein disulfide isomerases
PERK: protein kinase RNA (PKR)-like ER kinase
Phogrin: phosphatase homolog of granules from rat insulinomas
PTM: post-translational modification
PTPN22, protein tyrosine phosphatase: non-receptor type 22
ROS: reactive oxygen species
RyR: ryanodine-receptor
SERCA: sacro/endoplasmic reticulum Ca^2+^ ATPase
Tgase2: tissue transglutaminase 2
T1D: type 1 diabetes
UPR: unfolded protein response
XBP-1: X-box binding protein 1
ZnT8: zinc transporter 8.
